# Preoperative induction chemotherapy with cisplatin and irinotecan for pathological N_2_ non-small cell lung cancer

**DOI:** 10.1038/sj.bjc.6600117

**Published:** 2002-02-12

**Authors:** H Date, K Kiura, H Ueoka, M Tabata, M Aoe, A Andou, T Shibayama, N Shimizu

**Affiliations:** Department of Surgery II, Okayama University School of Medicine, 2-5-1 Shikata-cho, Okayama 700-8558, Japan; Department of Internal Medicine II, Okayama University School of Medicine, 2-5-1 Shikata-cho, Okayama 700-8558, Japan; Department of Internal Medicine, Tsuyama Central Hospital, 1756 Kawasaki, Tsuyama 708-0841, Japan

**Keywords:** cisplatin, irinotecan, induction chemotherapy, non-small cell lung cancer

## Abstract

We conducted a phase I/II study to investigate whether the surgical resection after induction chemotherapy with cisplatin and irinotecan was feasible and could improve the treatment outcome for patients with pathological N_2_ non-small cell lung cancer. Fifteen patients with stage IIIA non-small cell lung cancer having mediastinal lymph node metastases proved by mediastinoscopy were eligible. Both cisplatin (60 mg m^−2^) and irinotecan (50 mg m^−2^) were given on days 1 and 8. Patients received two cycles of chemotherapy after 3–4 weeks interval. Induction was followed by surgical resection in 4–6 weeks. Patients who had documented tumour regression after preoperative chemotherapy received two additional cycles of chemotherapy and other patients received radiotherapy postoperatively. After the induction chemotherapy, the objective response rate was 73%. All the 15 patients received surgical resection and complete resection was achieved in 11 (73%) patients. There was no operation-related death and one death due to radiation pneumonitis during postoperative radiotherapy. The median time from entry to final analysis was 46.5 months, ranging from 22 to 68 months. The 5-year survival rate was 40% for all the 15 patients and it was 55% for the 11 patients who underwent complete resection. We conclude that the surgical resection after induction chemotherapy with cisplatin and irinotecan is feasible, and associated with low morbidity and high respectability.

*British Journal of Cancer* (2002) **86**, 530–533. DOI: 10.1038/sj/bjc/6600117 www.bjcancer.com

© 2002 Cancer Research UK

## 

The prognosis for non-small-cell lung cancer (NSCLC) with metastases in mediastinal nodes after surgery is generally poor because distant micrometastases are frequently present. If these nodes are found before operation by mediastinoscopy and treated with surgical resection and postoperative irradiation, the 5-year survival is only approximately 10% (Pearson *et al*, 1982). Although many studies have been performed to evaluate adjuvant chemotherapy, the results of these studies have been disappointing on the whole. Recently, several neoadjuvant treatment schedules using induction chemotherapy (Roth *et al*, 1998; Rosell *et al*, 1999) or chemoradiotherapy (Rusch *et al*, 1993; Albain *et al*, 1995; Sugarbaker *et al*, 1995; Mathisen *et al*, 1996; Rice *et al*, 1998) followed by surgical resection have shown encouraging results.

We have recently reported the results of combination chemotherapy of cisplatin (CDDP) and irinotecan (CPT-11) for unresectable NSCLC (Ueoka *et al*, 1999). The objective response rate was 76% and the toxicity was manageable. The purpose of this study was to assess toxicity, response, and survival in patients with pathological N2, stage IIIA NSCLC who received induction chemotherapy with CDDP and CPT-11.

## MATERIALS AND METHODS

### Patient selection

Initial staging evaluation included complete history and physical examination, a complete blood cell count, standard chemistry profile, urinalysis, 24-h urine creatinine clearance, electrocardiogram, bronchoscopy, arterial blood gas analysis and pulmonary function test. Pretreatment chest X-ray, bone scan and CT of brain, chest and abdomen were required to ensure the absence of any haematogenous dissemination.

Eligibility requirements for entry into the study were as follows: (1) histologically or cytologically proven NSCLC (excluding bronchoalveolar carcinoma); (2) no prior chemotherapy, radiotherapy or surgery; (3) age of 75 years or less; (4) clinical N2, stage IIIA (T1–3) based on CT scanning; (5) pathological N2 proved by mediastinoscopy; (6) performance status (PS) of 0–1 on the Eastern Cooperative Oncology Group (ECOG) scale (Oken *et al*, 1982); (7) adequate pulmonary function to undergo surgical resection (predicted postoperative forced expiratory volume in 1 s per predicted preoperative forced vital capacity >0.3); (8) adequate functions of the kidney (creatinine clearance >60 ml min^−1^), liver (ALT, AST <twice the upper limit of normal), and bone marrow (a leukocyte count ⩾4000 μl^−1^ and a platelet count ⩾100 000 μl^−1^); (9) no other primary cancer within the 5 years before diagnosis and (10) a written form of informed consent.

Lymphadenopathy was initially investigated by CT scanning and mediastinal lymph nodes larger than 10 mm along their long axis (except for subcarinal nodes) were defined as metastatic lymph nodes (clinical N2). For subcarinal lymph nodes, nodes larger than 10 mm along their short axis were defined as metastatic nodes (clinical N2). These patients with clinical N2 then underwent a staging cervical mediastinoscopy with evaluation of bilateral node stations 2 and 4 and subcarinal station 7 as defined by The Japan Lung Cancer Society. An anterior mediastinoscopy with biopsy of station 5 or 6 was not done. Only patients with pathologically documented N2 (pathological N2) were eligible.

### Treatment plan

Planned treatment began with induction chemotherapy. Both CDDP and CPT-11 were given by 1-h infusion on days 1 and 8. Patients received two cycles of chemotherapy after 3–4 weeks interval. CDDP (60 mg m^−2^) was given with 100 ml of physiological saline just after administration of odansetron (4 mg) or granisetron (3 mg). CPT-11 (50 mg m^−2^) dissolved in 300 ml physiological saline was given after the administration of CDDP. After administration of CDDP and CPT-11, hydration consisting of 3000 ml of physiological saline was given. Toxicity was evaluated according to the criteria of ECOG (Oken *et al*, 1982). If grade 4 haematological toxicity or grade 3 non-haematological toxicity such as diarrhoea was observed in the previous course, the dose of CPT-11 was reduced by 10 mg m^−2^ in the next cycle. The dose of CDDP was reduced by 10 mg m^−2^ for development of grade 4 haematological toxicity or by 30 mg m^−2^ for development of grade 3 renal toxicity. Before the next course was started, leukocyte and platelet counts had to be at least 3000 μl^−1^ or more and 75 000 μl^−1^ or more respectively. If the leukocyte count was <2000 μl^−1^ or neutrophil count was <1000 μl^−1^ , granulocyte colony-stimulating factor (G-CSF) was administered subcutaneously at a dose of 50 μg m^−2^ until recovery.

Two to 4 weeks after the completion of induction chemotherapy, patients were restaged and evaluated for clinical response by chest X-ray, bone scan, and CT of brain, chest and abdomen. Progressive disease was defined as greater than 25% increase in the sum of the products of the crossed diameters of the primary lesion over the size present at study entry or the appearance of new locoregional or metastatic disease. Stable disease was defined as a change in the size of measured lesions between a 50% reduction and a 25% increase with no additional disease discovered. Partial response was defined as a reduction of greater than 50% in the sum of the products of the crossed diameters of the primary lesion and the absence of new lesions. Complete response was defined at the disappearance of all measurable intrathoracic disease.

Patients with progressive disease were taken off study and all other patients with response or stable disease underwent thoracotomy 4 to 6 weeks after the last dose of chemotherapy. The surgical procedure was based on the extent of tumour at the time of initial presentation rather than at the time of thoracotomy. Lobectomy was the preferred resection; however, if primary tumour invasion required, a sleeve resection, bilobectomy or pneumonectomy was done. Resection and reconstruction of the chest wall was also performed if necessary. A complete ipsilateral superior mediastinal lymphadenectomy was performed in all cases (resection of ipsilateral nodal stations 1, 2, 3, 4 and 7). For patients with primary lower lobe lesions, stations 8 and 9 lymph nodes were also resected. Patients with primary left lung lesions also had resection of stations 5 and 6 lymph nodes. The bronchial stump was covered with pedicled intercostal muscle or pericardial fat tissue. Complete resection was defined as a surgical resection in which the tumour and accessible mediastinal lymph nodes were completely removed, and incomplete resection was defined as a surgical resection in which the tumour, metastatic lymph nodes, or both were incompletely removed.

For patients who had documented a tumour reduction of greater than 50% after induction chemotherapy, two additional cycles of chemotherapy using an identical regimen were given starting 4 to 6 weeks after thoracotomy. Other patients received postoperative radiation therapy, 2.0 Gy a day, to a total dose of 50 Gy as an option.

### Follow-up

After the completion of all treatment, patients were evaluated in an outpatient clinic every month for a year, and every 3 months thereafter. Chest X-ray was taken at each visit. Bone scan and CT of brain, chest, and abdomen were required every 6 months for a year, and yearly thereafter or when clinical signs of recurrence developed. Recurrences were classified as locoregional (inside the ipsilateral thorax), distant (outside the ipsilateral thorax), or both. Survival curves were constructed using the Kaplan–Meier method. Survival times were calculated from the date of mediastinoscopy. Results were analyzed as of 28 February, 2001.

## RESULTS

Between June 1995 and April 1999, 411 patients with NSCLC received surgery in our centre. Among them, 15 patients (3.6%) with stage IIIA NSCLC having mediastinal lymph node metastases proved by mediastinoscopy were entered into this clinical trial. The distribution of patients entered in the trial by years is as follows; 1995, six patients; 1996, three patients; 1997, five patients; 1999, one patient. Characteristics of all patients are listed in Table 1

**Table 1:**
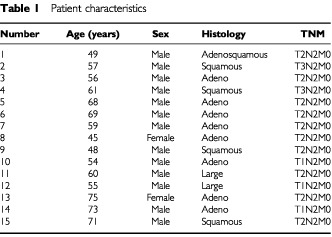
Patient characteristics

. The mean age was 60 years ranging from 45 to 75. There were 13 men and two women. Histological cell type of the final pathology specimen showed eight patients had adenocarcinoma, four had squamous, two had large-cell, and one had adeno-squamous cell lung cancer. The primary tumour at initial presentation was classified clinically as T1 in three, T2 in 10 and T3 in two. The diameter of the primary tumour ranged from 12 to 88 mm with an average of 43.3 mm. The size of the largest mediastinal lymph node along its long axis by CT ranged from 10 to 60 mm with an average of 26.1 mm. All patients had histologically proved N2 disease by cervical mediastinoscopy. Single nodes only were affected in five patients and 10 patients were found to have multiple positive nodes at the time of mediastinoscopy.

Toxicity from induction chemotherapy is summarized in Table 2

**Table 2:**
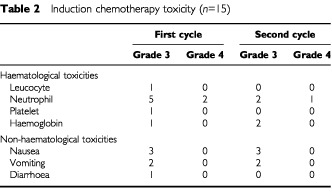
Induction chemotherapy toxicity (*n*=15)

. Haematological toxicity was generally mild. Two patients developed grade 4 neutropenia during the first cycle of chemotherapy and one patient during the second cycle. Non-haematological toxicity was entirely manageable without treatment interruption although nausea, vomiting and diarrhoea were experienced by most patients. There was no evidence of severe pulmonary, cardiac, hepatic or renal toxicity. Eleven patients (73%) received two cycles of chemotherapy without dose modification and four patients (27%) required dose modification due to toxicity.

After induction chemotherapy and before the surgical procedure, a clinical response determination was made on the basis of the second chest CT scan. At this time, 11 patients (73%) achieved a partial response and four patients (27%) had stable disease. No complete responses were seen and no patients had disease progression.

All the 15 patients underwent thoracotomy 4 to 6 weeks after the last dose of chemotherapy. Eleven patients (73%) had a complete surgical resection. Four patients (27%) had an incomplete resection (two had subcarinal nodes invading to the carina, and two had paratracheal nodes invading to the trachea and superior *vena cava*). No patient had an exploratory thoracotomy. Nine patients were treated with a lobectomy, three with a bilobectomy, one with pneumonectomy, one with a sleeve lobectomy and one with pneumonectomy and chest wall resection. One patient (7%) developed a bronchopleural fistula and empyema after right upper lobectomy. This complication was successfully managed by a chest tube drainage and pleural irrigation. Of note was that the stump of the bronchus was not covered at resection only in this patient. No other significant postoperative complication was encountered.

Three patients (20%) were judged to have no residual tumour in the primary site pathologically. Of the three patients, one patient (7%) had no evidence of tumour in any examined lymph nodes (T0N0M0) and another had only N1 diseased nodes (T0N1M0). Therefore, two patients (13%) were downstaged to mediastinal node negative disease at resection.

Among 11 patients who had shown partial response by induction chemotherapy, eight patients received two additional cycles of adjuvant chemotherapy using an identical regimen. Toxicity findings during adjuvant chemotherapy were similar to those during induction. Another three patients (two due to incomplete resection and one due to chest wall resection) received postoperative radiation therapy to a total dose of 50 Gy. Among four patients who had shown no response by induction chemotherapy, two received postoperative radiation therapy, one refused further treatment, and one did not receive any treatment due to bronchial fistula.

One right pneumonectomy patient died on postoperative day 74 from radiation pneumonitis which occurred after receiving a total dose of 20 Gy. This single death (7%) was considered treatment related.

At the time of final data analysis in February 2001, the median time from entry to final analysis for all patients was 46.5 months, ranging from 22 to 68 months. Median follow-up of patients alive was 48.6 months. Six patients (40%) were alive, two with recurrent disease. Of nine (60%) deaths, eight were from recurrent disease and one was from treatment. Locoregional failure occurred in three patients (20%) who underwent incomplete resection. Distant recurrence developed in seven patients (47%).

**Figure 1:**
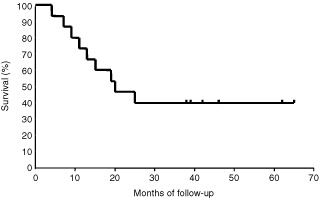
The Kaplan–Meier survival curve of all 15 patients. The median survival is 20 months with a projected survival of 40% at 5 years.

The projected 5-year Kaplan–Meier overall survival was 40%, with a median overall survival of 20 months ( Figure 1

). The 5-year survival for the 11 patients having complete resection was 55%, whereas, none of the four patients having incomplete resection survived for more than 20 months.

## DISCUSSION

Operation is accepted as the best therapy for stage I and II non-small cell lung cancer (NSCLC), achieving cure rates between 40 and 80%. The group of patients with affected ipsilateral mediastinal lymph nodes represents a challenge. It is critically important to stage these patients accurately before making treatment decisions. Although the chest CT scan is valuable to determine the extent to the primary disease, its assessment of the mediastinal lymph nodes might be falsely positive in as many as 30% of cases and falsely negative in 20%. All patients in our series had mediastinoscopy-positive cancers, which insures the assignment of these patients to the poor prognosis subgroup in stage IIIA.

There is a growing body of date regarding the multimodality induction approach to stage IIIA lung cancer. Two recent phase III trials of induction chemotherapy followed by resection in patients with stage IIIA disease, including some with T3N0 or T3N1 disease, have reported an improved survival in combined modality group (Roth *et al*, 1998; Rosell *et al*, 1999). These two studies lend credence to the concept of induction therapy for stage IIIA NSCLC. To date, the optimal regimen has yet to be defined and questions remain regarding whether chemotherapy is better alone or in combination with radiotherapy. We chose CDDP and CPT-11 as induction chemotherapeutic regimen on the bases of our previous experience in treating 17 patients with unresectable NSCLC (Ueoka *et al*, 1999). CPT-11 is a semisynthetic derivative of camptothecin that exerts its cytotoxic activity by inhibiting a nuclear enzyme topoisomerase I as a novel therapeutic target (Hsiang and Liu, 1988). The synergistic effect between CDDP and CPT-11 might be intensified because these drugs were simultaneously given for 2 days within one course. The recommended dose of CDDP and CPT-11 was determined to be 60 and 50 mg m^−2^. We avoided concurrent radiation therapy because chemotherapy with CDDP and CPT-11 plus concurrent radiation therapy was associated with unacceptable morbidity (Yokoyama *et al*, 1998).

The overall morbidity and mortality rates were quite acceptable. Haematological toxicity was generally mild. Non-haematological toxicity was entirely manageable without treatment interruption although nausea, vomiting and diarrhoea were experienced by most patients. There were no deaths from toxicity during induction chemotherapy. All patients fully recovered from these toxicities within 4–6 weeks and were able to receive surgical resection. Regarding postoperative complications, one patient (7%) developed a bronchopleural fistula and empyema. This complication was successfully managed by a chest tube drainage and pleural irrigation. We elected to cover the bronchial stump with pedicled viable tissue except this particular patient. One patient died from radiation pneumonitis after right pneumonectomy. This single death (7%) was considered treatment related. A 43% mortality after pneumonectomy was reported in an induction chemoradiotherapy trial (Fowler *et al*, 1993).

The objective radiographic response rate of 73% and the resectability of 73% of all eligible patients in this study agree with the previous trials of induction chemoradiotherapy (Rusch *et al*, 1993; Sugarbaker *et al*, 1995; Mathisen *et al*, 1996; Rice *et al*, 1998). However, pathological sterilization of mediastinal lymph nodes was seen only in two patients (13%). Induction chemoradiotherapy has achieved 40–46% incidence of pathological downstaging.

The ultimate test of any induction therapy rests on the survival rate. Our study includes only 15 patients, however, the median follow-up of 46.5 months was long enough to assess the long-term survival rate. The 5-year survival rate of 40% and median survival of 20 months was comparable to or slightly better than those seen in other studies that used induction chemoradiotherapy. The excellent 5-year survival of 55% in 11 patients who underwent complete resection suggests that complete surgical resection still play a substantial role on long-term survival in this stage of disease.

In conclusion, the surgical resection after induction chemotherapy with CDDP and CPT-11 is feasible, and associated with low morbidity and high respectability.
